# *MDM2* Oncogene Copy Number Alterations in Chronic Lymphocytic Leukemia

**DOI:** 10.4274/tjh.galenos.2019.2019.0179

**Published:** 2019-08-02

**Authors:** Pathum Sookaromdee, Viroj Wiwanitkit

**Affiliations:** 1 TWS Medical Center, Bangkok, Thailand; 2 Dr. DY Patil University, Pune, India

**Keywords:** MDM2, Oncogene, Copy number, Chronic lymphocytic leukemia

## To the Editor,

We read “Investigation of *MDM2* oncogene copy number alterations in cases of chronic lymphocytic leukemia” (CLL) with great interest [1]. Darbaş et al. [1] stated: “In previous studies, *MDM2* overexpression was examined at mRNA and protein levels, but amplification of the *MDM2* gene at DNA level in CLL patients has been examined for the first time in our study” [1]. Indeed, *MDM2* overexpression is an important pathological phenomenon seen in CLL [2]. The investigation of *MDM2* overexpression can be useful in the diagnostic and therapeutic management of the patient. Nevertheless, we would like to point out that the present report by Darbaş et al. [1] is not the first report studying amplification of the *MDM2* gene at DNA level in CLL patients. A previous report by Watanabe et al. [3] already investigated this issue, although in those case the results were negative.

## References

[1] Darbaş Ş, Aydın Ç, Salim O, Berker Karaüzüm S (2019). Investigation of MDM2 oncogene copy number alterations in cases of chronic lymphocytic leukemia. Turk J Hematol.

[2] Bixby D, Kujawski L, Wang S, Malek SN (2008). The pre-clinical development of MDM2 inhibitors in chronic lymphocytic leukemia uncovers a central role for p53 status in sensitivity to MDM2 inhibitor-mediated apoptosis. Cell Cycle.

[3] Watanabe T, Ichikawa A, Saito H, Hotta T (1996). Overexpression of the MDM2 oncogene in leukemia and lymphoma. Leuk Lymphoma.

