# Supplementary material for: MDM2 Oncogene Copy Number Alterations in Chronic Lymphocytic Leukemia
Source: Turk J Haematol. 2019 Aug 2;36(3):220–1. doi: 10.4274/tjh.galenos.2019.2019.0179 (PMC6682777; doi:10.4274/tjh.galenos.2019.2019.0179)
Supplement: Related texts

# Reply from the Authors

## To the Editor,

We would like to thank the authors for showing interest in our letter, which was titled “Investigation of *MDM2* oncogene copy number alterations in cases of chronic lymphocytic leukemia” [1]. The authors have shown two previously published articles related to overexpression of *MDM2* in CLL patients. One of them was written by Watanabe et al. [2]; they performed Southern blot analysis to examine whether *MDM2* was amplified in 23 B-CLL specimens and found that none of the patients showed amplification. In the other study, Bixby et al. [3] reported that they had found three copies of the *MDM2* gene in 37 of 178 CLL patients using 50K SNP-array technology. After screening the literature in detail, we found two additional studies. Bueso-Ramos et al. [4] reported 5 CLL patients not showing amplification using the Southern blot method and Huang et al. [5] reported 4 of 11 patients with CLL showing amplification of the *MDM2* gene using the Southern blot technique. Considering these four studies, amplification of the *MDM2* gene at DNA level in CLL patients had been previously examined by Southern blot and array techniques, but we used the FISH technique in our study to demonstrate *MDM2* amplification in CLL patients.

## References

[1] Darbaş Ş, Aydın Ç, Salim O, Berker Karaüzüm S (2019). Investigation of MDM2 oncogene copy number alterations in cases of chronic lymphocytic leukemia. Turk J Hematol.

[2] Watanabe T, Ichikawa A, Saito H, Hotta T (1996). Overexpression of the MDM2 oncogene in leukemia and lymphoma. Leuk Lymphoma.

[3] Bixby D, Kujawski L, Wang S, Malek SN (2008). The pre-clinical development of MDM2 inhibitors in chronic lymphocytic leukemia uncovers a central role for p53 status in sensitivity to MDM2 inhibitor-mediated apoptosis. Cell Cycle.

[4] Bueso-Ramos CE, Yang Y, deLeon E, McCown P, Stass SA, Albitar M (1993). The human MDM-2 oncogene is overexpressed in leukemias. Blood.

[5] Huang YQ, Raphael B, Buchbinder A, Li JJ, Zhang WG, Friedman-Kien AE (1994). Rearrangement and expression of MDM2 oncogene in chronic lymphocytic leukemia. Am J Hematol.
